# Effectiveness of the Internet Attachment-Based Compassion Therapy (iABCT) to improve the quality of life and well-being in a population with chronic medical illness: A study protocol of a randomized controlled trial (SPIRIT compliant)

**DOI:** 10.1371/journal.pone.0278462

**Published:** 2022-12-27

**Authors:** Marian Martínez-Sanchis, Mª Dolores Vara, Rocío Herrero, Daniel Campos, Javier García-Campayo, Rosa Mª Baños

**Affiliations:** 1 Polibienestar Research Institute, University of Valencia, Valencia, Spain; 2 Department of Personality, Evaluation and Psychological Treatment, Faculty of Psychology, University of Valencia, Valencia, Spain; 3 CIBER-Obn Physiopathology of Obesity and Nutrition, Instituto de Salud Carlos III, Madrid, Spain; 4 Department of Psychology and Sociology, Faculty of Humanities and Educational Sciences, University of Zaragoza, Huesca, Spain; 5 Instituto de Investigación Sanitaria de Aragón (IIS Aragón), (IIS Aragón), Zaragoza, Spain; 6 Primary Care Prevention and Health Promotion Research Network, RedIAPP, Madrid, Spain; 7 Department of Medicine, Psychiatry and Dermatology, University of Zaragoza, Zaragoza, Spain; 8 Hospital Universitario Miguel Servet, Zaragoza, Spain; Centre de Recherche Scientifique et Technique sur les Regions Arides, ALGERIA

## Abstract

**Background:**

Chronic medical illnesses significantly and negatively affect the quality of life of individuals who suffer them and represent one of the most important challenges faced by healthcare providers and policy-makers due to its rising prevalence and high rates of comorbidity. Compassion-based interventions delivered over the Internet may be a useful approach to facilitate illness management and improve the quality of life of individuals with chronic medical conditions.

**Objectives:**

The purpose of this study is to describe a protocol for a randomized controlled trial to test the efficacy of the Internet Attachment-Based Compassion Therapy (iABCT) to improve the quality of life and well-being of patients with chronic medical illnesses.

**Method:**

A two-arm, parallel-group, randomized controlled trial (RCT) will be carried out, with three assessment points (baseline, 3-month, and 6-month) under two conditions: intervention group and control group (waiting list). The primary outcomes include the quality of life on the EuroQol 5-Dimensions Questionnaire (EQ-5D) and the Pemberton Happiness Index (PHI). Secondary outcomes, such as compassion, self-care behaviors, illness interference, self-criticism, symptomatology, attachment styles, social support, and illness perception, will be considered. Moreover, an assessment on satisfaction and usability will be carried out. A total of 68 participants as minimum will be recruited (34 per arm). Intent-to-treat mixed-model analyses without any ad hoc imputations will be conducted.

**Conclusions:**

Findings of this study will provide new insights into the potential of self-applied compassion-based interventions (CBI) delivered online in the context of chronic medical illnesses, considering aspects of their implementation (e.g., facilitators, barriers) and mechanisms of change.

**Trial registration:**

The study is registered under Clinicaltrials.gov (NCT04809610) and it is currently in the participant recruitment phase.

## Introduction

Chronic illnesses are defined as non-communicable diseases that last one year or more, require ongoing treatment or suppose a significant functional alteration or both [1, 2]. Due to its rising prevalence along with their important consequences (e.g., hospitalization, long-term disability or work limitations), these conditions are becoming a significant burden not only to individuals who suffer them, but also their families and society [3–5].

Furthermore, there are high rates of comorbidity between mental health problems and chronic medical conditions, being one of the most important challenges faced by health-care providers and policymakers [6, 7]. These comorbidities have been extensively reviewed [8, 9], and studies have shown that individuals with chronic medical illnesses (e.g., chronic pain, diabetes or inflammatory bowel disease) commonly experience depression or anxiety [10–12], and negative health outcomes such as low satisfaction with life [13] or diminished quality of life [14]. This comorbidity reflects a reciprocal interaction in which the negative consequences of the disease not only depend on the course and severity of the illness itself, but also on other psychological processes. For instance, the psychological adjustment process to the disease, the coping strategies to face stress associated with the disease, and the patient’s illness management have a direct influence on physical health, including morbidity, mortality and disease complications [15–18]. Likewise, detrimental psychological processes (e.g., illness-related shame, self-criticism or rumination) suppose a barrier to implement self-care behaviors involved in an adaptive illness management (e.g., treatment plan adherence, exercising, or dietary guidelines adherence), which, in turn, could further increase distress, worsen prognosis and detriment the quality of life [19–22]. As a consequence, both self-care behaviors included in the illness management as well as detrimental psychological processes have become a target of different psychological interventions aimed at promoting quality of life of the population with chronic medical illness [23].

This is the case of compassion-based interventions (CBI), which refer to psychological interventions aimed at enhancing compassionate and self-compassionate responses which involve the recognition of suffering and the inclination to relieve it with an act of kindness rather than criticizing, blaming, or pitying [24]. Compassion and self-compassion may be a useful approach to illness management since they involve accepting suffering as an inevitable human condition and facilitate a kinder and more compassionate attitude towards difficulties [25].

Several studies have shown that CBIs can be especially beneficial for improving the quality of life of people with chronic illness [26–28]. Two recent systematic reviews have concluded that CBIs are effective in populations with chronic medical conditions, as their results showed improvements in different physical and psychological outcomes such as depression, anxiety, self-compassion and health-related quality of life [29, 30].

In sum, being able to approach difficulties with a compassionate attitude helps individuals to feel empowered with new management strategies and promote a sense of calm and agency to provide comfort to themselves, which facilitates the implementations of self-care behaviors and adaptive illness management.

One of the CBI that has proved its efficacy on both healthy population and patients with chronic medical conditions is the Attachment-Based Compassion Therapy (ABCT) [31]. This therapy is based on attachment theory [32], which provides a framework for understanding the links between close relationships and psychopathology and includes specific practices to identify and develop a secure attachment style to promote compassion for oneself and others [33]. Specifically, the ABCT has shown its efficacy and applicability for the treatment of fibromyalgia showing improvements on psychological outcomes such as functional status [34] and biological outcomes such as reduced brain-derived neurotrophic factor (BDNF) and inflammatory biomarkers [35]. There are specific delivery barriers that could interfere with the effectiveness of CBIs in people with chronic illnesses, such as limitations of access, mobility, or transportation. In order to tackle these limitations and to respond to the growing need of healthcare systems for scalability and sustainability, evidence-based interventions can benefit from adapting its delivery format through information and communications technologies (ICTs) [36, 37]. In fact, previous research supports the notion that the psychological outcomes of people living with chronic illnesses can be improved with a self-delivered online intervention (e.g. [38, 39]) and incipient evidence shows the potential value of delivering CBIs online in the context of chronic illnesses [40, 41]. Moreover, other results show that adapting CBIs to an online format may improve adherence and facilitate the involvement of chronic patients in a better management of their illness [37]. Regarding the ABCT approach, an online version (iABCT) has been developed to be totally self-applied over the Internet for Spanish speakers. The iABCT is currently be assessed in a feasibility study for general population [42].

In conclusion, CBIs delivered through the Internet seem to be a helpful cost-effective solution, and incipient research is exploring the feasibility and acceptability of the iABCT on a Spanish general population [42]. Therefore, more research is needed on their efficacy in a context of chronic medical conditions. The present study is the first attempt to explore the efficacy of the iABCT to improve quality of life and well-being in this population.

### Objectives

The main goal of this study is to describe a randomized controlled trial (RCT) to test the efficacy of the iABCT [42] to improve the quality of life and well-being in a population with chronic medical illness, compared to waiting list (WL) as a control condition.

In particular, this study has seven specific objectives: (1) to analyze the efficacy of the iABCT program in improving quality of life and well-being and other secondary outcomes (compassion, self-compassion, self-care behaviors, illness interference, self-criticism, symptomatology, attachment styles, social support, illness perception) at 3-month follow-up after baseline compared to the WL group; (2) to provide 6-month follow-up data on the maintenance or generalization of the achievements in the intervention group; (3) to analyze the acceptability of the iABCT program in terms of expectations, satisfaction, usability and opinion by the participants; (4) to provide 6-month follow-up data for the control group after receiving the iABCT; (5) to analyze which patient profile (i.e., sociodemographic and clinical baseline variables) predicts the improvement in quality of life and well-being after receiving iABCT in the follow-up sessions; (6) to analyze which mechanism of change predicts the greatest change in measures related to quality of life and well-being in the follow-up sessions; and (7) to identify possible facilitators and barriers to the use of this type of protocols (e.g., treatment logic, duration, format, usability) taking into account the participant’s perspective through a semi-structured qualitative interview.

We hypothesize that: (a) iABCT condition will be more efficacious than the WL group in improving quality of life and well-being and other secondary variables at 3-month follow-up; (b) the gains achieved after iABCT will be maintained at 6-month follow-up; and (c) the iACBT will be well accepted in terms of expectations, satisfaction, usability and opinion by the participants; (d) participants in WL group will show an improving quality of life and well-being and other secondary variables at a 6-month follow-up, once they receive the iABCT; (e) improvements in quality of life and well-being scores will be predicted by self-reported self-compassion and secure attachment; (f) frequency of meditation practice will moderate the improvement in quality of life and well-being after receiving the iABCT; and (g) changes in compassion, self-criticism and attachment style will appear as mediators of greatest changes in quality of life and well-being. Regarding objective 7, no specific hypotheses are proposed due to the exploratory nature of the qualitative analyses.

## Material and methods

### Research design

A two-arm, parallel-group, randomized controlled trial (RCT) will be carried out to determine the effectiveness of the iABCT on improving the quality of life and well-being of a chronic medical illness population by comparing individuals who have access to the intervention with a WL control group.

Participants will be randomly assigned to one of the two conditions: a) intervention group, and b) control group (WL) and will complete different measures at different assessment points: baseline time (pre-assessment), and 3- and 6-month follow-up. Participants of the WL group will receive access to the iABCT once they fulfill the 3-month assessment.

### Study setting and recruitment

Participants will be recruited from April 2021 to April 2022 by several means, including the study website and flyers posted on social media platforms (e.g., Facebook, Instagram, LinkedIn), doctors’ referrals, and through chronic illness patients’ associations. All distributed materials will include a dedicated email address through which participants could contact the research team for further information on the study.

### Eligibility criteria

Participants will be selected according to the following eligibility criteria:

#### Inclusion criteria

(1) Age > 18 years; (2) ability to understand and read Spanish; (3) access to a computer with the Internet; and (4) diagnosis of one of the following chronic medical conditions: diabetes, inflammatory bowel disease, fibromyalgia, low-back chronic pain, migraines and other conditions.

#### Exclusion criteria

(1) Terminal disease; (2) severe psychiatric disorders comorbidities (schizophrenia, substance dependence, bipolar disorder, psychotic illness) or severe neurologic or medical condition; and (3) receiving psychological treatment or mindfulness training at the time of recruitment.

### Intervention

#### Experimental group. Internet attachment-based compassion therapy (iABCT)

The iABCT is an online self-applied version of Attachment-based Compassion Therapy (ABCT) that has been adapted and optimized to be delivered via the Internet [42]. As mentioned, the ABCT is based on the attachment theory and the use of compassion meditations, and therefore includes practices to raise awareness and/or address maladaptive aspects (when appropriate) of attachment styles developed with parents and compassion and self-compassion practices to improve interpersonal relationships and well-being general [31]. Following the original version, the iABCT is composed of 8 modules that have been formulated to be completely self-applied and share the same structure: (1) module objectives; (2) theoretical contents of the module; (3) exercises and activities (including formal and informal practices) to put what is learned in the module into practice; (4) assessment of the knowledge acquired during the module; (5) tasks to be completed before advancing to the next module (homework assignments); and (6) summary of the module. Formal and informal compassion and self-compassion meditations such as receiving and giving compassion to oneself, friends, unknown people, and people deemed to be problematic; identifying their own attachment style; and understanding how it influences their current interpersonal relationships, are practiced during the intervention. The content of the modules includes texts, images, illustrations, videos, audio-guided meditations, interactive exercises, and daily homework assignments. Downloaded PDF files will be made available so that users can review them offline. Each module has been optimized to have a duration of approximately 60 or 90 minutes. The entire intervention is estimated to be completed in eight weeks. For more information about the iABCT, see Refs [42].

#### Control group. Waiting list (WL) control group

The control group will receive no intervention for the first three months after enrolling in the study and complete the first assessment (pre-assessment). After completing the second assessment (3 months), participants in this condition will be informed that they will have access to the iABCT program.

### Strategies for monitoring and improving intervention protocol adherence

In order to improve adherence, email reminders will be sent after 7 days if participants have not started with the pre-assessment (for both groups) or the treatment modules (for the intervention group) yet. These reminders will contain messages encouraging them to start with the assessment or intervention (in the case of the intervention group) and asking them for any difficulties or technical problems to enter the web platform. Moreover, an email at the midpoint of the intervention (module 3) will be sent to those participants who have not progressed through the intervention. In this case, messages will include positive reinforcement to the participants, reminders of the importance of reviewing the modules and encouraging messages to do the homework tasks and continue with the iABCT program.

### Data collection and outcomes

Participants will be assessed at baseline (pre-assessment) and 3- and 6-month follow-up.

**Fig 1:**
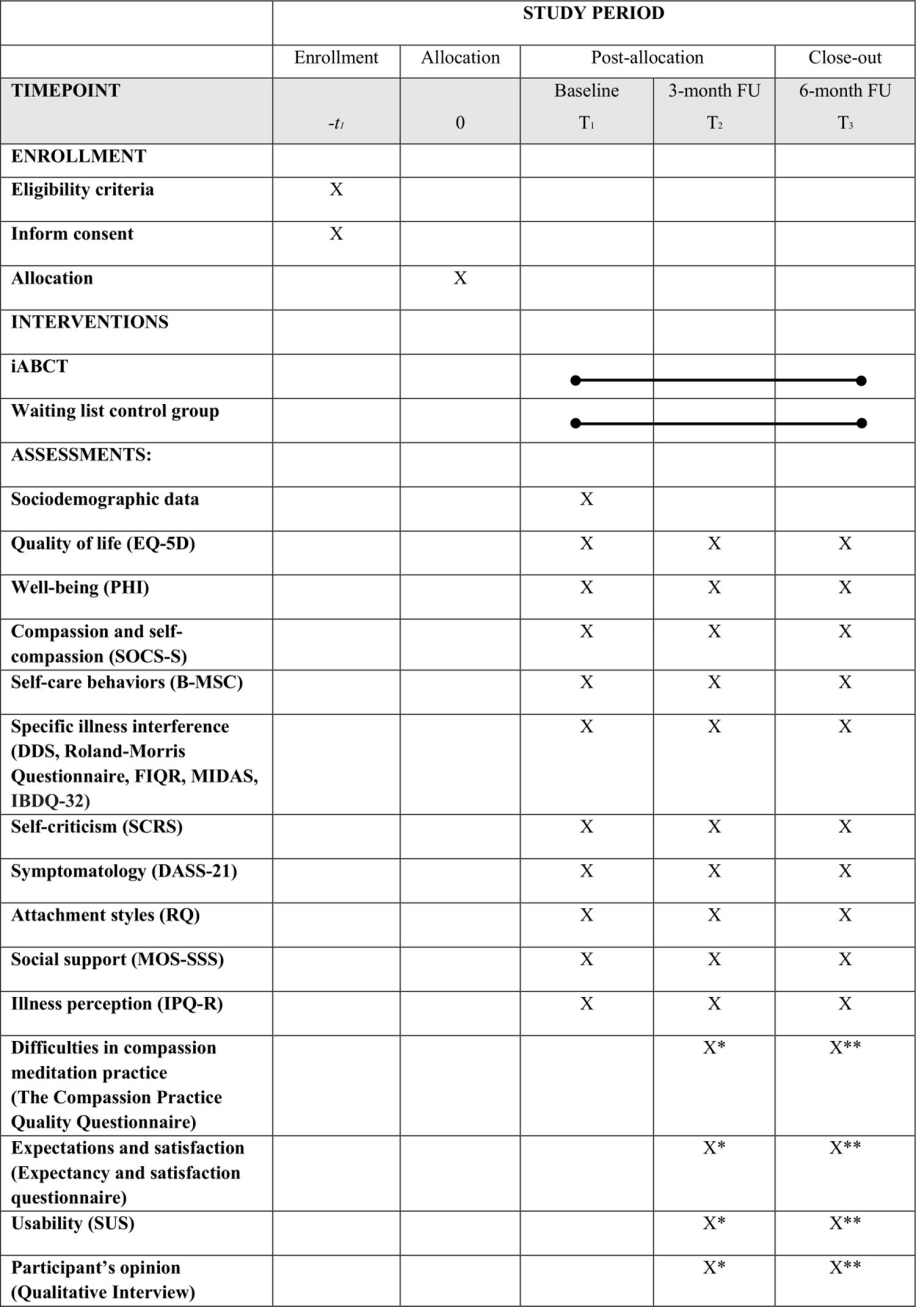
SPIRIT flow diagram: Schedule of enrolment, interventions and assessments.

Assessments will be conducted online via the platform web (https://psicologiaytecnologia.labpsitec.es/), where the iABCT is hosted. Outcome assessment will follow the schedule outlined in Fig 1.

#### Screening related measures

*Sociodemographic variables and meditation practice*. To ensure that eligibility criteria are met, information regarding diagnosis of any mental or physical condition will be collected and participants will be asked if they are receiving a psychological intervention or a training based on the practice of meditation prior to being enrolled in the study.

Demographic data (age, sex, nationality, educational level, occupation, and civil status) will be collected as part of the baseline assessment (pre-assessment). Variables regarding overall meditation practice will be recorded as follows: any or no meditation experience, source of learning (i.e., self-taught, therapy context, teacher or secular training course, and religious context), frequency of meditation (daily, 3 or 4 times a week, once a week or less, 2 or 3 times per month, sporadically, never), duration of each session (mean time in minutes), and lifetime practice (in years), and for participants with experience, the amount of time (in months) of meditation practice interruption and context of practice (secular or religious).

#### Primary outcome measure

*Quality of life*. The EuroQol 5-Dimensions Questionnaire (EQ-5D; [43]) is a self-report measure that assesses the health-related quality of life (HRQOL) using five dimensions of functionality in daily-life: mobility, self-care, usual activities, pain/discomfort and anxiety/depression. and three levels of severity. Responses in each dimension are divided into three levels of severity: (1) no problems; (2) moderate problems; and (3) extreme problems. Moreover, the EQ-5D includes a visual analogue scale (EQ VAS) in which participants self-rate their quality of life in a range from 0 (worst imaginable health state) to 100 (best imaginable health state). The Spanish version of this scale has shown good reliability and validity properties [44].

*Well-being*. The Pemberton Happiness Index (PHI; [45]) is a scale which includes 11 items related to different domains of remembered well-being (general, hedonic, eudaimonic, and social well-being) and ten items related to experienced well-being (i.e., positive and negative emotional events that possibly happened the day before); the sum of these items produces a combined well-being index that ranges from 0 to 10. Results from the Spanish validation study provided very good support for the psychometric properties of the PHI (α = .92) ([45]).

#### Secondary outcome measures

*Compassion and self-compassion*. The Sussex-Oxford Compassion for the Self Scale (SOCS-S; [46]) is a 20-item scale that assesses the levels of self-compassion in a 5-point Likert scale ranging from 1 (not at all true) to 5 (always true). It includes 5 subscales: recognizing suffering, understanding the universality of suffering, feeling for the person suffering, tolerating uncomfortable feelings, and acting or being motivated to act to alleviate suffering. The validation study of this scale has shown adequate psychometric properties (Cronbach’s alphas ranged from .75 to .93 for total SOCS-S scale and subscale items) [46]. For this study a translated and adapted version of the original scale into Spanish will be used.

*Self-care behaviors*. The Mindful Self-Care Scale—Brief version (B-MSC; [47]) is the 24-item shortened version of the Mindful Self Care Scale (MSCS; [48]). This scale measures the frequency of self-care behaviors addressed in 6 domains: mindful relaxation, physical care, self-compassion and purpose, supportive relationships, supportive structure, and mindfulness. Cronbach’s alpha for the B-MSCS domains showed adequate values ranging from .74 to .86 [47]. In this study, a translated and adapted version of the original scale into Spanish will be used.

*Specific illness interference measures*. Specific questionnaires will be used to assess specific illness interferences for the different medical conditions included in the study: the Diabetes Distress Scale (DDS; [49]) for participants with type I and type II diabetes; the Roland-Morris Questionnaire [50] for participants with low-back chronic pain; the Revised Fibromyalgia Impact Questionnaire (FIQR; [51]) for participants with fibromyalgia; the Migraine Disability Assessment questionnaire (MIDAS; [52]) for participants with migraines; and the Inflammatory Bowel Disease Questionnaire (IBDQ-32; [53]) for participants with inflammatory bowel illness.

*Self-criticism*. The Self-critical Rumination Scale (SCRS; [54]) is a 10-item questionnaire measuring self-criticism, defined as a form of negative thinking that devalues the self. It assesses thoughts that criticize the self for perceived errors, failures, weaknesses, defects, bad habits, or general inadequacy. It takes into account the ruminative qualities of thought: frequency, duration, repetition, and difficulty of control. Final scores range from 10 to 40 and higher scores indicate a higher level of self-criticism. The Spanish validation of this scale has shown good psychometric properties (α = .91) [55].

*Symptomatology*. The Depression, Anxiety and Stress Scale (DASS-21; [56]) is an abbreviated form of the DASS questionnaire containing 21 items and organized in three subscales to assess anxiety, depression, and stress symptomatology. Responses indicate the presence of each symptom over the past week on a 4-point Likert-type scale ranging from 0 (*never*) to 3 (*almost always*). The Spanish validation of this scale has shown acceptable internal consistency (Cronbach’s alpha for the total score was 0.94 and Cronbach’ alpha for Depression, Anxiety and Stress scales were 0.85, 0.85 and 0.87, respectively) [57].

*Attachment styles*. The Relationships Questionnaire (RQ; [58]) uses a 7-point Likert-scale that assesses and matches participants with one of four attachment styles: (i) secure, (ii) preoccupied, (iii) dismissive and (iv) fearful. Studies for both original and Spanish versions of the scale have demonstrated that the reliability of the self-descriptor criteria is high [59].

*Social support*. The Medical Outcomes Study-Social Support Survey (MOS-SSS; [60]) is a 20-item questionnaire that analyzes the perception of social support. Item 1 refers to the size of the social network and the remaining 19 items measure four dimensions of functional social support: emotional (8 items), instrumental (4 items), positive social interaction (4 items), and affective support (3 items). The items present a 5-point response scale that measures progressively (from never to always) how often each type of social support is available to the caregiver. A total score can also be obtained, where higher scores would indicate more support received. The Spanish version of the scale has shown good reliability properties (α = .94) [61].

*Illness perception*. The Illness Perception Questionnaire-Revised (IPQ-R; [62]) was designed to assess the cognitive and emotional dimensions of illness representations. The IPQ-R consists of three sections. Two scales measure the identity and causal dimensions, and another general section assesses the dimensions of duration (acute/chronic), cyclical course, consequences, personal control, treatment control, coherence, and emotional representations. Cronbach’s alpha values in the Spanish validation study showed acceptable psychometric properties, ranging from .39 to .81 for the different subscales [63].

*Difficulties in compassion meditation practice*. The Compassion Practice Quality Scale (CPQS) [64] has been developed to assess the key aspects of compassion practices (e.g., mental imagery, sense of connection and warmth, compassionate phrases, and compassionate gestures). This self-reported questionnaire includes two subscales (imaginery and somatic perception) with a total of 10 items that participants score on a scale ranging from 0 to 100 indicating the percentage of the time that their experience reflects each statement. Higher scores indicate higher quality of practice (i.e., less practice difficulties). Both subscales (imagenery and somatic) have been shown to be reliable, with Cronbach’s alpha values of .90 and .88 respectively [64].

#### Acceptability outcome measures of intervention

*Expectations and satisfaction*. Expectancy and satisfaction questionnaire (adapted by Campos et al., 2020 [42] from Borkovec and Nau, 1972 [65]). These questionnaires were adapted from Borkovec and Nau (1972) [65] to measure participant expectations before the intervention and their later satisfaction with it. Each scale includes six items rated from 0 ("not at all") to 10 ("very much"). The questions addressed how logical the intervention seemed, to what extent the participant expected to be satisfied with it, whether the participant would recommend the intervention to others, whether it would be useful for psychological problems, the interventions’ usefulness for the participants, and to what extent it could be aversive (reverse item). Higher scores represent higher expectations and satisfaction levels.

*Usability*. System Usability Scale (SUS; adapted by Campos et al., 2020 [42]). This instrument was adapted from the System Usability Scale (SUS) in order to assess the usability of a service or product and the acceptance of technology by the people who use it. The SUS has been shown to be a valuable and robust tool for assessing the quality of a wide range of user interfaces, as it is easy to use and understand. This scale includes 10 statements rated on a five-point scale measuring agreement with the statement (0 = strongly disagree; 4 = strongly agree). The final score is obtained by adding the scores on each item and multiplying the result by 2.5. Scores range from 0 to 100, where higher scores indicate better usability.

*Participant’s opinion*. ***Semi-structured qualitative interview*.** A qualitative interview will be adapted to explore reasons for participant’s dropout or no participation in the trial [66, 67]. Reasons for dropping out or not participating in the trial will be further explored using an online form with pre-set response options, and multiple reasons will be allowed. Additionally, options will be available to expand participants’ qualitative responses. A final three open questions will explore the general opinion of the program.

### Participant timeline

Interested participants will receive an access link to fill in the written informed consent together with the screening questionnaire through the LimeSurvey platform (https://www.limesurvey.org/es/). The eligibility assessment will consist of demographic questions, assessment of history and current state of mental health and treatment history, and type or chronic medical illness diagnosis. After consenting, eligible participants will be identified with a code to which only the researcher will have access. They will then be registered on the Psychology and Technology web platform (https://psicologiaytecnologia.labpsitec.es/) to complete the assessment measures at different points in time: pre-assessment and follow-up assessments. Participants in the intervention condition will receive an email after intervention completion to get their feedback on the program (e.g., ease of use, barriers, possible improvements).

Participants in the WL group will be able to access the web platform for the intervention at 3 months (after the active treatment group).

### Sample size

The sample will be composed of 68 participants as minimum. The sample size was calculated using the G*Power 3.1.9.7 for Windows program [68]. Estimated sample size was decided based on medium effect sizes (d = 0.4), statistical power of .80 and α = .05, indicating a necessary sample of 52 participants [69]. Considering that the dropout rate in Internet-based interventions for the chronic medical ill population is usually around 30%, a target of 34 people per group was set [70–72].

### Allocation and blinding

After giving online written informed consent to participate in the study and completing online eligibility and baseline questionnaires, participants who meet the criteria will be randomized to the experimental or the WL control group. An allocation scheme will be created by a member of the research team blind to the study using a computerized random generator (random allocation software 2.0) at a 1:1 ratio. Randomization will be stratified by type of chronic medical condition (diabetes, fibromyalgia, intestinal inflammatory illness, migraines, low-back chronic pain and other conditions). After randomization is completed, both researcher and participant will be informed with the results. Participants in the intervention groups will have access to the program right after receiving the outcome, meanwhile participants in the WL control group will have to wait 3 months to have access to the intervention. As both participants and researcher will be informed about which of the two conditions participants are assigned, blinding for the treatment is not possible.

### Statistical methods

The latest version of SPSS (v.26) will be used for all data analysis. T-tests for independent-samples (t), ANOVAs, and chi-square analysis (χ2) will be calculated to investigate the characteristics of the randomized sampled and included in the RCT and to explore group differences in participants’ sociodemographic (e.g., age, gender) and clinical data (e.g., quality of life, well-being). Attrition and dropout rates will be calculated by reporting percentages and patterns of missing data. Intent-to-treat mixed-model analyses without any ad hoc imputations will be used to handle missing data in case of participant dropout and to conduct the efficacy analysis [73]. This analysis compares the study groups based on the treatment to which they were randomly allocated. It does not assume that the last measurement is stable, it does not involve any substitution of missing values with supposed or estimated values, it is conducted using all available observations, thus reducing the biases and loss of power caused by the simple deletion or random imputation of incomplete data. Mixed-model analyses are appropriate for RCTs with multiple time points and pre-post designs, and it has showed remarkable robustness to violations of distributional assumptions (e.g., skewed, kurtosis, or heteroscedastic) [74, 75]. To study distributional assumptions and the impact of missing random effect components on model some test will be included: Normality assumptions will be explored using the Shapiro-Wilk test (for each experimental group, N < 50), the Kolmogorov-Smirnov (K-S) test (for the total sample, N > 50), skewness and kurtosis indices, and normality plots (Q-Q plots). Homoscedasticity will be explored by Levene’s test and Box’s M test for equivalence of covariance matrices. Wald statistic (or Z-test) will be conducted to test the residual error variance estimation and the null hypothesis of homogeneity of residuals. The assumption that data is missing completely at random (MCAR) will be evaluated using Little’s MCAR test. it is robust to violations of distributional assumptions. A linear mixed-effect model for each outcome measure will be implemented using the MIXED procedure with one random intercept per subject. An identity covariance structure will be specified to model the covariance structure of the intercept. For each outcome, time (baseline and 3- and 6-month follow-up) will be treated as a within-group factor and group (experimental and control group) as a between-group factor and significance effects will be followed up with pairwise comparisons (adjusted by Bonferroni correction). Cohen’s d effect size and 95% CI will be calculated for within- and between-group comparisons [76, 77]. Sensitivity analyses will be performed to assess the robustness of the findings in terms of different methods for handling missing data (i.e., mixed models with and without imputation, maximum-likelihood estimation, and maximum-likelihood multiple imputation [78]. The means and standard deviations of the acceptability measures of the intervention will be analyzed. For analyses of associations between intervention outcomes, predictors of change and mechanisms of action, several statistical tests such as Pearson’s correlation, multiple regressions and mediation analysis will be performed. In addition, participants’ qualitative responses regarding facilitators and barriers to the intervention will be explored using a qualitative content analysis and coding and categorization approach to the data using word frequency counts with ATLAS.ti software.

The state of the art of analytic methodology for RCT will be reviewed before analyzing the data, in order to apply the most appropriate statistical analysis procedure.

### Data management

Online data collected from LimeSurvey and Psychology and Technology platforms will be stored at protected servers of the University of Valencia. Only individuals authorized by the principal investigator will have access to the database. After completing the assessment points, authorized individuals will download the database in a SPSS format that will be automatically saved onto a password-protected cloud server of the University of Valencia (nÚVol, https://nuvol.uv.es).

Database will be stored at online secured servers from the University of Valencia for up to 5 years after the project ends.

### Data monitoring

The research team will be monitoring the proper conduction of the trial and participant safety. During the RCT, participant’s interest will be overseen and safeguarded, the overall conduct of the trial will be monitored, and participants’ safety will be ensured by systematically checking negative events and reacting to any extreme distress. Additional help and counseling will be strongly recommended to participants in the case of any adverse event or emergency.

Periodical meetings will be conducted by the research team after the three assessment points (pre-assessment and follow-up) to ensure the proper progress of the study and the current state of the data collection.

Important protocol modifications will be communicated to relevant parties (i.e., trial participants, trial registries, ethical committee, and researchers).

### Harms

So far, no studies have reported harms with CBIs. In case participants identify signs of mental or physical health detriment during participation, they will be provided with a list of health resources available and strongly recommended to visit their usual health professional.

### Auditing

No audit has been planned at this time.

### Research ethics approval, protocol amendments and consent

This trial will be conducted in compliance with the study protocol, the Declaration of Helsinki, and good clinical practice. Ethical approval for this trial has been obtained for the Ethics Committee of Research in Humans of the Ethics Commission in Experimental Research of University of Valencia (UV-INV_ETICA-1564960) on March 5, 2021.

The study was registered under Clinicaltrials.gov (NCT04809610) and will be conducted following the Standard Protocol Items: Recommendations for Interventional Trials (SPIRIT) guidelines [79]. The SPIRIT checklist was used as a guide for reporting this study protocol.

Prior to participation, interested participants will have access to the written informed consent online form, which will include details of the trial, explanation about potential risks and benefits and contact information of the research members. Participants will know that the intervention is completely optional and can be discontinued at any time. Written informed consent will be obtained online by pressing the consent button when the participant agrees to participate in the trial, before any intervention and assessments are provided.

### Confidentiality

Data security/confidentiality will be guaranteed according to Spanish Organic Law 3/2018 of December 5 on the Protection of Personal Data and Guarantee of Digital Rights and all relevant EU and Spanish privacy laws will be observed and respected.

Access to the web platform will be granted via a unique username-password combination. Data collected via the website will be stored on secure servers at University of Valencia (UVEG) with personal data and user-generated data stored in separate databases on different servers. The consent form will be explained and required from all participants at the eligibility assessment.

Data will be kept for a maximum of 5 years after the end of the project and participants will have the right to access, rectify personal information, withdraw consent and request deletion of their personal data.

### Access to data

Prior to the publication of the results, only data managers and principal investigators will have access to the database, will address any problem related to the data and finalize a dataset for statistical analysis.

After publication, data will be accessible on a digital platform following the procedure stated by fair guideline principle.

### Ancillary and post-trial care

Participants will be able to contact the researchers during the trial period or until 3 months after the end of participation. Although serious adverse events are not expected, if any participant develops any adverse event, they will be responded to promptly and appropriately even after study completion.

### Dissemination policy

Results from this trial will be published and disseminated in a peer-reviewed journal and public presentations at scientific and clinical conferences. Moreover, participation at important national and international academic conventions will be delivered and results will be shared to the general population and interested stakeholders via social media to further promote dissemination of the results.

## Results

Enrollment started in April 2021 and will be finished in April 2021. Data analysis will start in January 2022.

## Discussion

The objective of this paper is to describe the study protocol of a RCT designed to explore the efficacy of the iABCT on a population with chronic medical illness.

Recent evidence has highlighted the training of compassion and self-compassion as an effective approach to chronic medical illness [29, 30]. Moreover, online adaptations of these treatments suppose a highly-accessible and sustainable mode of delivery which improves psychological outcomes among people living with these medical conditions [38, 41].

Although promising studies have been aimed at exploring the adaptation of CBI to an online format on populations with chronic medical illnesses [40, 41], research in this context is still scarce and deeper exploration is needed. To our knowledge, this is the first attempt to explore the efficacy of the iABCT on improving the quality of life in a population with chronic medical illnesses.

The approach of the iABCT is based on the attachment styles theory [31]. The aim of this intervention is to build a secure attachment style through the training of compassion and self-compassion, which has been considered as a key therapeutic factor [31, 80]. It is expected that those improvements will help individuals with chronic conditions to approach their illness and related difficulties with a kind and compassionate response. This, in turn, would promote self-care behaviors and improve their illness management, thus leading to a higher quality of life and well-being.

Furthermore, since different aspects of the intervention implementation and mechanisms of change will be investigated, this study has potential to shed light on the relationships between the different constructs assessed together with compassion and self-compassion outcomes (e.g., self-criticism, attachment styles) and to extend our current understanding of barriers and facilitators of CBIs delivered online in the context of chronic medical illness.

This trial has some limitations that require consideration. First, since the control group in the current study is a waitlist control rather than active control, relative utility of the iABCT will not be explored. Future studies could benefit from including an active control to explore relative utility of the modality (e.g. comparing Internet version of the ABCT with the original treatment) or the treatment approach (e.g. comparing iABCT with iCBT). Secondly, since stratification will not be balanced, some comparative analysis will not be possible to explore if sample size of each group is not sufficient and equal to each group (e.g. comparisons regarding specific illness interference across different types of chronic medical conditions).

Despite these limitations, findings from this study will add valuable data to the incipient research field on the potential of self-applied CBIs via the internet in the context of chronic medical illnesses [40, 41].

## Supporting information

S1 File SPIRIT checklist. (PDF)

S1 File Ethics committee protocol. (PDF)
