# Supplementary material for: Effectiveness of the Internet Attachment-Based Compassion Therapy (iABCT) to improve the quality of life and well-being in a population with chronic medical illness: A study protocol of a randomized controlled trial (SPIRIT compliant)
Source: PLoS One. 2022 Dec 27;17(12):e0278462. doi: 10.1371/journal.pone.0278462 (PMC9794054; doi:10.1371/journal.pone.0278462)
Supplement: S1 File — (PDF) [file pone.0278462.s002.pdf]

El comité Ético de Investigación en Humanos de la Comisión de Ética en Investigación Experimental de la Universitat de València,

CERTIFICA:

Que el Comité d'Ètica d'Investigació en Humans, en la reunión celebrada el día 04 de Marzo de 2021, una vez estudiado el proyecto de tesis doctoral: *"Eficacia de una intervención online y auto-aplicada centrada en la terapia de compasión basada en los estilos de apego para mejorar la calidad de vida en población con enfermedad médica crónica"*,
Cuyo/a responsable es D/Dña.
ROSA MARIA BAÑOS RIVERA, dirigida por D/Dña. ROSA MARIA
BAÑOS RIVERA
ha acordado informar favorablemente el mismo.

Y para que conste, se firma el presente certificado

Av. Blasco Ibáñez, 13 tel: 963864109 vicerec.investigacio@uv.es
València 46010 fax: 963983221 www.uv.es/serinves

Firmado digitalmente por
PEDRO JESUS PEREZ ZAFRILLA
Cargo: Presidente del Comité de Ética de la Investigación en Humanos
Fecha: 05/03/2021 00:09:16 CET

The Ethics Committee of Research in Humans of the Ethics Commission in Experimental Research of University of Valencia,

CERTIFY:

Hereby certify that the Ethics Committee of Research in Humans, in the session which took place on marzo 04, 2021, analysed the project of doctoral thesis entitled *"Efficacy of an online and self-applied intervention focused on compassion therapy based on attachment styles to improve the quality of life in a population with chronic medical illness"*, whose researcher in charge is ROSA MARIA BAÑOS RIVERA, and agreed with this project.

And in witness whereof, I hereby sign this certificate

Comité d'Ètica d'Investigació en Humans de la Comissió d'Ètica en Investigació Experimental de la Universitat de València,

CERTIFICA:
Que Comité d'Ètica d'Investigació en Humans, en la reunió que tingué lloc el dia 04 de març de 2021, una vegada estudiat el projecte de tesi doctoral titulat:*"Eficàcia d'una intervenció en línia i auto-aplicada centrada en la teràpia de compassió basada en els estils d'aferrament per millorar la qualitat de vida en població amb malaltia mèdica crònica"*,
que té com a responsable
ROSA MARIA BAÑOS RIVERA, i que va dirigir ROSA MARIA BAÑOS RIVERA,
ha acordat emetre'n un informe favorablement.

I perquè així conste, signa aquest certificat.

Av. Blasco Ibáñez, 13 tel: 963864109 vicerec.investigacio@uv.es
València 46010 fax: 963983221 www.uv.es/serinves
